# Domestic *Triatoma sanguisuga*–Human Exposure in the South Carolina Coastal Region

**DOI:** 10.4269/ajtmh.20-0043

**Published:** 2020-08-03

**Authors:** Kyndall C. Dye-Braumuller, Chris L. Evans, Mary K. Lynn, Colin J. Forsyth, Claudia Gomez, Melissa S. Nolan

**Affiliations:** 1 Laboratory of Vector-Borne and Zoonotic Diseases, Arnold School of Public Health, University of South Carolina, Columbia, South Carolina;; 2 South Carolina Department of Health and Environmental Control, Bureau of Environmental Health Services, Columbia, South Carolina;; 3 Chagas Treatment Access Project, Drugs for Neglected Diseases Initiative-North America, New York, New York;; 4 Vector-Transmitted Diseases, Department of Santander, Colombia

## Abstract

A collaborative investigation was initiated in rural coastal South Carolina in response to a reported triatomine bite. The eastern conenose bug, *Triatoma sanguisuga*, was identified and tested for *Trypanosoma cruzi*. The insect was negative by PCR, and no additional triatomines were found in the vicinity of the home. This is the first published report of a bite from *T. sanguisuga* in South Carolina despite the fact that triatomine vectors have been documented in the state since the 1850s, and specimens have been collected from homes in the past. Sylvatic *T. cruzi* reservoirs are common throughout the southeastern United States, and this case brings to light the possibility of human contact with infected triatomines in the state of South Carolina for public health and clinical and entomology professionals.

In June 2019, a 51 year-old Hispanic woman was sitting on a bed leaned against a wall in an upstairs bedroom when she felt something crawling on her upper left arm. She discovered four insect bites and a triatomine insect still on her arm (later saved at room temperature in a bottle), Figure 1. The woman and her husband were visiting relatives living in Dorchester County, SC. The woman recognized the insect as the vector for the causative agent of Chagas disease from her home country of Colombia, but realized it was a different species as indicated by her comment that the current triatomine was “black with red markings instead of brown (like in Colombia),” Figure 2. After discussing the bite with the homeowners, the family requested assistance from local authorities. A collaborative investigation between the state public health entomologist, the University of South Carolina, and the CDC’s Division of Parasitic Diseases and Malaria was initiated.

**Figure 1.**
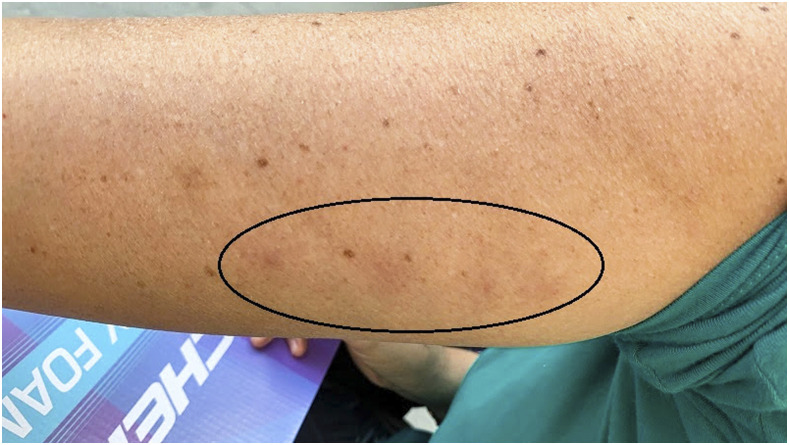
An image of the victim’s arm following the triatomine bites. Note the red areas (circled) where the skin is red and raised. This figure appears in color at www.ajtmh.org.

**Figure 2.**
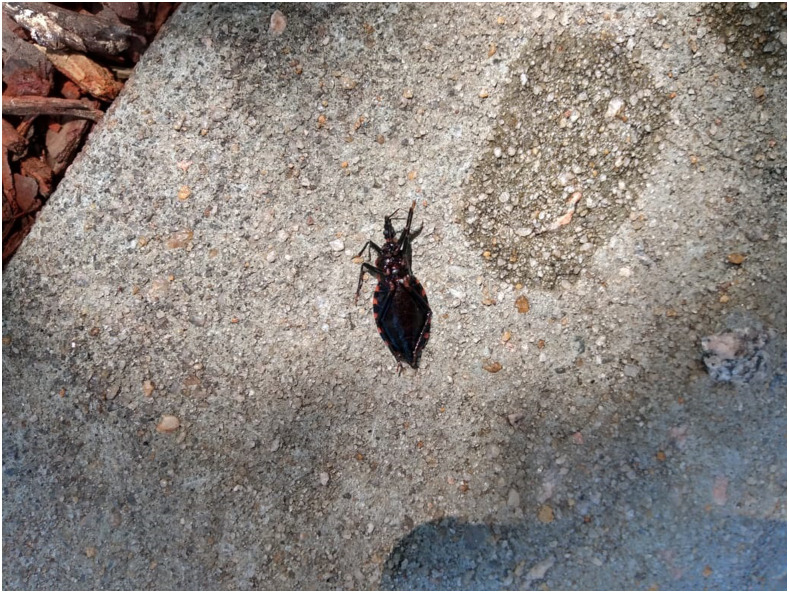
The triatomine immediately after it was discovered and killed by the house occupants. This is an image of the ventral side of the triatomine. This figure appears in color at www.ajtmh.org.

All house occupants (*n* = 5) screened negative for *Trypanosoma cruzi* antibodies by Chagas STAT-PAK^®^ (Chembio, Medford, NY); additional whole blood samples were not available for additional diagnostics. This was the first notable triatomine encounter according to the homeowners. Domestic opportunities for triatomine intrusion were considered low risk: home in good condition, “never” left doors or windows open, no exterior cracks, and no pets. Potential residential triatomine habitats included the home’s pier and beam foundation with lattice perimeter, the rural surrounding area with sylvatic animals present, and two large unused wood piles immediately adjacent to the homeowner’s property line, Figure 3. An additional investigation into these wood piles did not reveal any triatomines. However, these wood piles were noted as appropriate sites for rodents and marsupials, which are frequently colonized by triatomines in the United States and act as reservoir hosts for *T. cruzi*.^1^ Furthermore, two attempts to collect additional triatomines at the residence were executed, but both attempts were during or directly following heavy rain events, which are adverse collection conditions for triatomines.^2,3^ The bite-associated triatomine was morphologically identified as an adult female *Triatoma sanguisuga*, the eastern conenose bug, using a dichotomous key.^4^ The insect was *T. cruzi* DNA negative by PCR performed at the CDC’s Division of Parasitic Diseases and Malaria.

**Figure 3.**
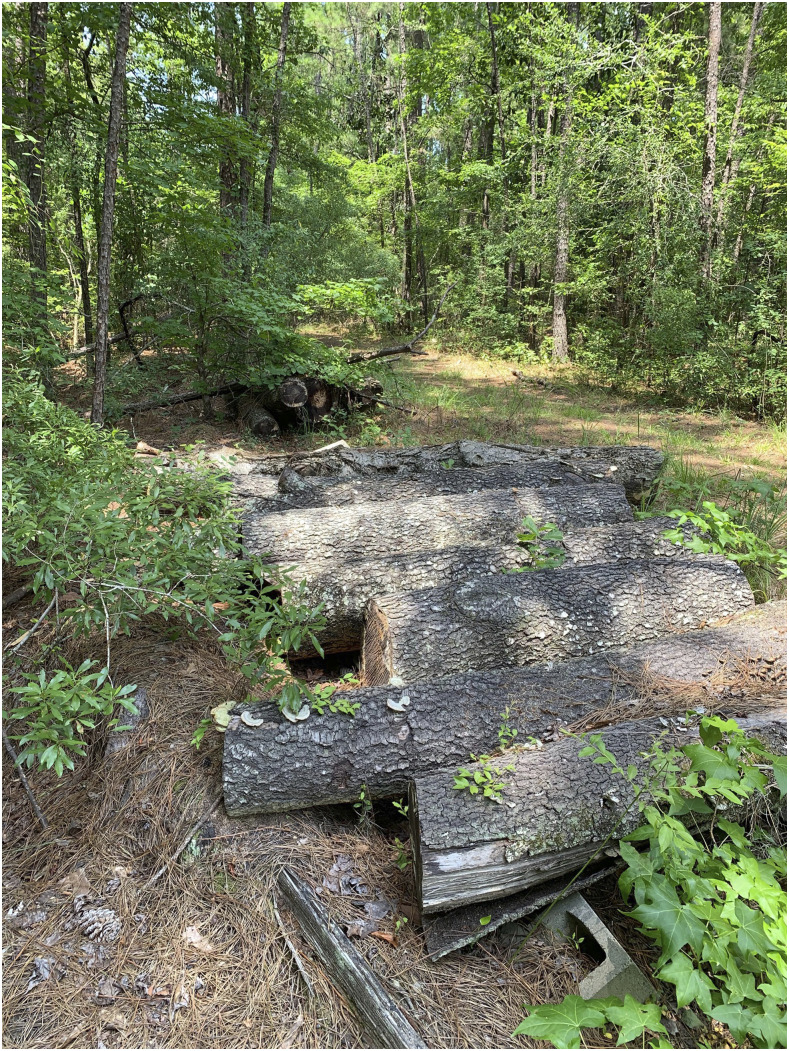
One of the large wood piles adjacent to the property line of the homeowner. These tree trunks provide an appropriate environment for various peri-domestic animals such as rodents and marsupials. This figure appears in color at www.ajtmh.org.

This finding is the first published case report of a *T. sanguisuga* bite in South Carolina. Approximately 35 *Triatoma* specimens from 14 counties are housed in state insect collections, dating back to 1934 (M. Ferro and M. Gibson, personal communication). Two *T. sanguisuga* from the Clemson Arthropod Collection were involved in biting humans in Hampton and Jasper counties in the 1990s, and one was found on a bed inside a domicile; however, *T. cruzi* testing was not performed (M. Ferro, personal communication). As recently as August 2019, three *T. sanguisuga* kissing bugs were submitted to the state health department from Jasper County, SC, with no reported bites to humans (C. L. Evans, unpublished data).

*Triatoma lecticularia* and *T. sanguisuga* are historically published vectors dating to 1859, when *T. lecticularia* was noted in “the Carolinas,”^5^ and one *T. lecticularia* was identified in Kershaw County, SC, in 1961 (Mike Ferro, personal communication). *Triatoma sanguisuga* has been identified in the state as recently as 2002 (M. Ferro, personal communication). Both *T. sanguisuga* and *T. lecticularia* have also been documented in the surrounding states: Georgia, North Carolina, and Tennessee in both domestic and peri-domestic environments.^1^ The fifth reported autochthonous case of Chagas disease in the United States was reported in neighboring rural Tennessee in 1999.^6^ *Triatoma sanguisuga* was implicated as the vector for this case; additional specimens were found around the patient’s home, as well as *T. cruzi*–positive raccoons and a dog.^6^

Sylvatic transmission has also been documented in South Carolina and surrounding states. Multiple studies have targeted raccoons for parasite detection; a relatively high percentage of these hosts have frequently tested positive for *T. cruzi* in Georgia,^7–12^ South Carolina,^12^ North Carolina,^13^ and Tennessee.^6,14^ A 2002 raccoon surveillance program in Georgia and South Carolina indicated animals captured in cities and coastal regions had statistically higher infection prevalence than those from rural and inland areas.^12^ Additional peri-domestic animals that have tested positive for *T. cruzi* in Georgia include opossums,^7,8,10,15^ striped skunks,^7,10^ gray foxes,^7^ coyotes,^10^ and bobcats.^10^ Wild opossums in North Carolina have also tested positive for the parasite.^13^ In addition, a 2007 study demonstrated a low *T. cruzi* prevalence in wild canids from SC Aiken and Barnwell counties.^16^ Overall, our report indicates that triatomines should be of interest to public health professionals in the southeastern U.S. coastal region, especially for those who move into sylvatic host animal habitats where risk for contact with infected vectors significantly increases.^1^

## References

[1] Bern C Kjos S Yabsley MJ Montgomery SP, 2011 *Trypanosoma cruzi* and Chagas’ disease in the United States. Clin Microbiol Rev 24: 655–681. 21976603 10.1128/CMR.00005-11 PMC3194829

[2] Ekkens DB, 1981 Nocturnal flights of *Triatoma* (Hemiptera: Reduviidae) in Sabino Canyon, Arizona: I. light collections. J Med Entomol 18: 211–227.

[3] Vazquez-Prokopec GM Ceballos LA Kitron U Gürtler RE, 2004 Active dispersal of natural populations of *Triatoma infestans* (Hemiptera: Reduviidae) in rural northwestern Argentina. J Med Entomol 41: 614–621. 15311452 10.1603/0022-2585-41.4.614 PMC1351236

[4] Lent H Wygodzinsky P, 1979 Revision of the Triatominae (Hemiptera, Reduviidae), and their significance as vectors of Chagas’ disease. Bull Am Mus Nat Hist 163: 123–520.

[5] Ryckman RE Ryckman JV, 1967 Epizootiology of *Trypanosoma cruzi* in southwestern North America Part XII: does Gause’s rule apply to the ectoparasitic Triatominae? (Hemiptera: Reduviidae) (Kinetoplastidae: Trypanosomidae) (Rodentia: Cricetidae). J Med Entomol 4: 379–386. 4963989 10.1093/jmedent/4.3.379

[6] Herwaldt B Grijalva M Newsome A McGhee C Powell M Nemec D Steurer F Eberhard M, 1999 Use of PCR to diagnose the fifth reported US case of autochthonous transmission of *Trypanosoma Cruzi*–Tennessee, 1998. Am J Trop Med Hyg 61: 564. 10.1086/315212 10608796

[7] McKeever S Gorman GW Norman L, 1958 Occurrence of a *Trypanosoma cruzi*-like organism in some mammals from southwestern Georgia and northwestern Florida. J Parasitol 44: 583–587. 13621315

[8] Pung OJ Banks CW Jones DN Krissinger MW, 1995 *Trypanosoma cruzi* in wild raccoons, opossums, and triatomine bugs in southeast Georgia, USA. J Parasitol 81: 324–326. 7707220

[9] Pietrzak SM Pung OJ, 1998 Trypanosomiasis in raccoons from Georgia. J Wildl Dis 34: 132–136. 9476234 10.7589/0090-3558-34.1.132

[10] Brown EL Roellig DM Gompper ME Monello RJ Wenning KM Gabriel MW Yabsley MJ, 2010 Seroprevalence of *Trypanosoma cruzi* among eleven potential reservoir species from six states across the southern United States. Vector-Borne Zoonotic Dis 10: 757–763. 20020815 10.1089/vbz.2009.0009 PMC2976638

[11] Schaffer G Hanson W Davidson W Nettles V, 1978 Hematotropic parasites of translocated raccoons in the southeast. J Am Vet Med Assoc 173: 1148–1151. 104938

[12] Yabsley MJ Noblet GP, 2002 Seroprevalence of *Trypanosoma cruzi* in raccoons from South Carolina and Georgia. J Wildl Dis 38: 75–83. 11838232 10.7589/0090-3558-38.1.75

[13] Karsten V Davis C Kuhn R, 1992 *Trypanosoma cruzi* in wild raccoons and opossums in North Carolina. J Parasitol 78: 547–549. 1597808

[14] Maloney J Newsome A Huang J Kirby J Kranz M Wateska A Dunlap B Yabsley MJ Dunn JR Jones TF, 2010 Seroprevalence of *Trypanosoma cruzi* in raccoons from Tennessee. J Parasitol 96: 353–358. 20001097 10.1645/GE-2312.1

[15] Parrish EA Mead AJ, 2010 Determining the prevalence of *Trypanosoma cruzi* in road-killed opossums (*Didelphis virginiana*) from Baldwin county, Georgia, using polymerase chain reaction. Ga J Sci 68: 132.

[16] Rosypal AC Tidwell RR Lindsay DS, 2007 Prevalence of antibodies to *Leishmania infantum* and *Trypanosoma cruzi* in wild canids from South Carolina. J Parasitol 93: 955–958. 17918387 10.1645/GE-1057R.1

